# Orbitale Blutung während eines Tauchgangs

**DOI:** 10.1007/s00347-020-01144-z

**Published:** 2020-06-22

**Authors:** Markus Gruber, Thomas Reinhard, Philip Maier

**Affiliations:** Klinik für Augenheilkunde, Universitätsklinikum Freiburg, Killianstr. 5, 79106 Freiburg, Deutschland

## Anamnese

Ein 55-jähriger Mann stellte sich, mit einem Trockentauchanzug gekleidet, aufgrund einer starken orbitalen Blutung in der Klinik für Augenheilkunde Freiburg vor. Während eines Tauchgangs habe er in etwa 40 m Tiefe plötzlich einen Knall wahrgenommen und einen stechenden Schmerz in der linken Orbita verspürt. Daraufhin habe sich die Taucherbrille teilweise mit Blut gefüllt. Glücklicherweise habe er Ruhe bewahren und ohne weitere Zwischenfälle auftauchen können. Das linke Auge sei im Alter von 5 Jahren aufgrund einer Pfeil-Bogen-Verletzung enukleiert worden. Zum Zeitpunkt des Tauchgangs habe er wie immer eine Glasprothese getragen.

## Klinische Untersuchung

**Figure 1:**
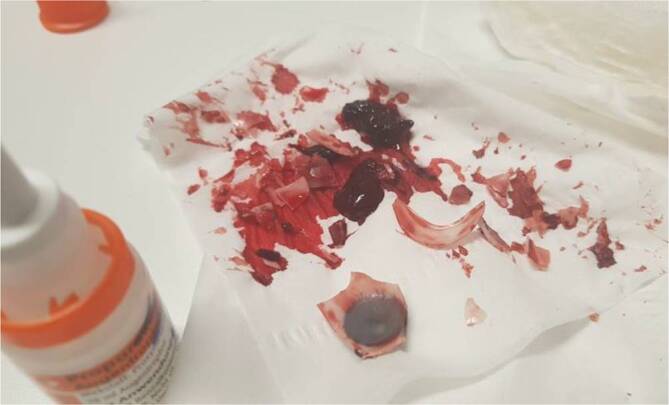

**Figure 2:**
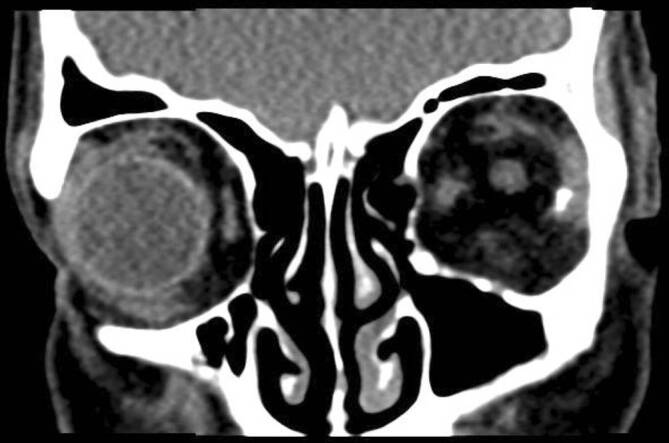

Links zeigten sich bei Zustand nach Enukleation makroskopisch erkennbare Blutkoagel und Glassplitter in der Orbita (Abb. 1). Die Lider waren unverletzt. Das rechte Auge zeigte bei einem Visus von 1,0 einen unauffälligen Spaltlampenbefund. Zur Abschätzung der Eindringtiefe der Glassplitter wurde eine Computertomographie durchgeführt. Es zeigten sich intraorbital zahlreiche Fremdkörper sowie die verbliebenen Augenmuskeln und der Sehnerv (Abb. 2). Frakturen der knöchernen Orbita waren nicht feststellbar.

## Wie lautet Ihre Diagnose?

## Therapie und Verlauf

Die Glassplitter wurden entfernt. Die Blutungen der Bindehaut hatten bereits sistiert. Eine Naht war nicht notwendig. Die Wunden der Bindehaut heilten unter Pflege mit Bepanthen- und Floxal-Augensalbe (Bayer, Leverkusen, Deutschland; Floxal, Bausch&Lomb, Rochester, New York, USA) innerhalb weniger Tage aus.

## Diskussion

Unserem Wissen nach handelt es sich bei dem vorliegenden Fall um den ersten Bericht einer Glasprothesenimplosion während eines Tauchgangs. Der älteste Nachweis einer Augenprothese datiert ca. 4800 Jahre zurück und stammt aus dem Iran [1]. Bitumen und Gold waren damals verwendete Materialien. Heutzutage werden in Europa wegen ihrer Kratzfestigkeit und Haltbarkeit hauptsächlich hohlwandige Prothesen aus Kryolith-Glas verwendet [2]. Diese zeichnen sich im Vergleich zu Polymethylmethacrylat(PMMA)-Prothesen durch geringeres Gewicht und höheren Tragekomfort aus. Unter extremen Umständen können Glasprothesen jedoch brechen und orbitale Schnittverletzungen verursachen. Patienten mit außergewöhnlichen Freizeitaktivitäten wie in diesem Fall sollten entsprechend beraten werden. Dabei kommen neben Kunststoffprothesen auch einwandige Glasprothesen infrage. Eine Abschätzung der möglichen Tauchtiefe mit doppelwandigen Prothesen ist aufgrund deren individueller Fertigung nicht möglich.

**Diagnose:** Implosion einer hohlwandigen Glasprothese aufgrund des Wasserdrucks
